# Supplementary material for: Thymol@activated Carbon Nanohybrid for Low-Density Polyethylene-Based Active Packaging Films for Pork Fillets’ Shelf-Life Extension
Source: Foods. 2023 Jul 3;12(13):2590. doi: 10.3390/foods12132590 (PMC10340350; doi:10.3390/foods12132590)
Supplement: Supplementary file 1 [file foods-12-02590-s001.zip › foods-2460868-supplementary.pdf]

Table S1. Data from thermogravimetric experiments used control release experiments of thymol from LDPE/5TO@AC film sample

LDPE/5TO@AC sample 1_average thickness 0.06mm			LDPE/5TO@AC sample 2_average thickness 0.06mm			LDPE/5TO@AC sample 3_average thickness 0.06mm		
**t**	**m_t/m_0**	**1-m_t/m_0**	**t**	**m_t/m_0**	**1-m_t/m_0**	**t**	**m_t/m_0**	**1-m_t/m_0**
0.00	1.00	0.00	0.00	1.00	0.00	0.00	1.00	0.00
60	0.98	0.02	60	0.98	0.02	60	0.98	0.02
120	0.98	0.02	120	0.98	0.02	120	0.98	0.02
180	0.98	0.02	180	0.98	0.02	180	0.98	0.02
240	0.98	0.02	240	0.98	0.02	240	0.98	0.02
300	0.98	0.02	300	0.98	0.02	300	0.98	0.02
360	0.98	0.02	360	0.98	0.02	360	0.98	0.02
420	0.98	0.02	420	0.98	0.02	420	0.98	0.02
480	0.98	0.02	480	0.98	0.02	480	0.98	0.02
540	0.98	0.02	540	0.98	0.02	540	0.98	0.02
600	0.98	0.02	600	0.98	0.02	600	0.98	0.02
660	0.98	0.02	660	0.98	0.02	660	0.98	0.02
720	0.98	0.02	720	0.98	0.02	720	0.98	0.02
780	0.98	0.02	780	0.98	0.02	780	0.98	0.02
840	0.98	0.02	840	0.98	0.02	840	0.98	0.02
900	0.97	0.03	900	0.98	0.02	900	0.98	0.02
960	0.97	0.03	960	0.98	0.02	960	0.98	0.02
1020	0.97	0.03	1020	0.98	0.02	1020	0.98	0.02
1080	0.97	0.03	1080	0.98	0.02	1080	0.98	0.02
1140	0.97	0.03	1140	0.98	0.02	1140	0.98	0.02
1200	0.97	0.03	1200	0.98	0.02	1200	0.98	0.02
1260	0.97	0.03	1260	0.98	0.02	1260	0.98	0.02
1320	0.97	0.03	1320	0.98	0.02	1320	0.98	0.02
1380	0.97	0.03	1380	0.98	0.02	1380	0.98	0.02
1440	0.97	0.03	1440	0.98	0.02	1440	0.98	0.02

Table S2. Data from thermogravimetric experiments used for control release experiments of thymol from LDPE/10TO@AC film sample.

LDPE/10TO@AC sample 1_average thickness 0.08mm			LDPE/10TO@AC sample 2_average thickness 0.07mm			LDPE/10TO@AC sample 3_average thickness 0.06mm		
**t**	**m_t/m_0**	**$1-m_t/m_0$**	**t**	**m_t/m_0**	**$1-m_t/m_0$**	**t**	**m_t/m_0**	**$1-m_t/m_0$**
0.00	1.00	0.00	0.00	1.00	0.00	0.00	1.00	0.00
60	0.98	0.02	60	0.98	0.02	60	0.97	0.03
120	0.97	0.03	120	0.98	0.02	120	0.96	0.04
180	0.97	0.03	180	0.98	0.02	180	0.96	0.04
240	0.97	0.03	240	0.98	0.02	240	0.96	0.04
300	0.97	0.03	300	0.98	0.02	300	0.96	0.04
360	0.97	0.03	360	0.98	0.02	360	0.96	0.04
420	0.97	0.03	420	0.97	0.03	420	0.96	0.04
480	0.97	0.03	480	0.97	0.03	480	0.96	0.04
540	0.97	0.03	540	0.97	0.03	540	0.96	0.04
600	0.97	0.03	600	0.97	0.03	600	0.96	0.04
660	0.97	0.03	660	0.97	0.03	660	0.96	0.04
720	0.97	0.03	720	0.97	0.03	720	0.96	0.04
780	0.97	0.03	780	0.97	0.03	780	0.96	0.04
840	0.97	0.03	840	0.97	0.03	840	0.96	0.04
900	0.97	0.03	900	0.97	0.03	900	0.96	0.04
960	0.97	0.03	960	0.97	0.03	960	0.96	0.04
1020	0.97	0.03	1020	0.97	0.03	1020	0.96	0.04
1080	0.97	0.03	1080	0.97	0.03	1080	0.96	0.04
1140	0.97	0.03	1140	0.97	0.03	1140	0.96	0.04
1200	0.97	0.03	1200	0.97	0.03	1200	0.96	0.04
1260	0.97	0.03	1260	0.97	0.03	1260	0.96	0.04
1320	0.97	0.03	1320	0.00	1.00	1320	0.96	0.04
1380	0.97	0.03	1380	0.00	1.00	1380	0.96	0.04
1440	0.00	1.00	1440	0.00	1.00	1440	0.96	0.04

Table S3. Data from thermogravimetric experiments used for control release experiments of thymol from LDPE/15TO@AC film sample.

LDPE/15TO@AC sample 1_average thickness 0.10mm			LDPE/15TO@AC sample 2_average thickness 0.09mm			LDPE/15TO@AC sample 3_average thickness 0.05mm		
**t**	**m_t/m_0**	**$1-m_t/m_0$**	**t**	**m_t/m_0**	**$1-m_t/m_0$**	**t**	**m_t/m_0**	**$1-m_t/m_0$**
0.00	1.00	0.00	0.00	1.00	0.00	0.00	1.00	0.00
60	0.96	0.04	60	0.96	0.04	60	0.97	0.03
120	0.96	0.04	120	0.96	0.04	120	0.97	0.03
180	0.96	0.04	180	0.96	0.04	180	0.97	0.03
240	0.96	0.04	240	0.96	0.04	240	0.97	0.03
300	0.95	0.05	300	0.96	0.04	300	0.97	0.03
360	0.95	0.05	360	0.96	0.04	360	0.97	0.03
420	0.95	0.05	420	0.96	0.04	420	0.97	0.03
480	0.95	0.05	480	0.96	0.04	480	0.97	0.03
540	0.95	0.05	540	0.96	0.04	540	0.97	0.03
600	0.95	0.05	600	0.96	0.04	600	0.97	0.03
660	0.95	0.05	660	0.96	0.04	660	0.97	0.03
720	0.95	0.05	720	0.96	0.04	720	0.97	0.03
780	0.95	0.05	780	0.96	0.04	780	0.97	0.03
840	0.95	0.05	840	0.96	0.04	840	0.97	0.03
900	0.95	0.05	900	0.96	0.04	900	0.97	0.03
960	0.95	0.05	960	0.96	0.04	960	0.97	0.03
1020	0.95	0.05	1020	0.96	0.04	1020	0.97	0.03
1080	0.95	0.05	1080	0.96	0.04	1080	0.97	0.03
1140	0.95	0.05	1140	0.96	0.04	1140	0.97	0.03
1200	0.95	0.05	1200	0.96	0.04	1200	0.97	0.03
1260	0.95	0.05	1260	0.96	0.04	1260	0.97	0.03
1320	0.95	0.05	1320	0.96	0.04	1320	0.97	0.03
1380	0.95	0.05	1380	0.96	0.04	1380	0.97	0.03
1440	0.94	0.06	1440	0.96	0.04	1440	0.97	0.03
1500	0.94	0.06	1500	0.00	1.00	1500	0.96	0.04
1560	0.94	0.06	1560	0.00	1.00	1560	0.96	0.04
1620	0.94	0.06	1620	0.00	1.00	1620	0.96	0.04
1680	0.94	0.06	1680	0.00	1.00	1680	0.96	0.04
1740	0.94	0.06	1740	0.00	1.00	1740	0.96	0.04
1800	0.94	0.06	1800	0.00	1.00	1800	0.96	0.04

Table S4. Multiple comparison tests of TBARS values of pork meat during storage with respect to packaging treatments

Multiple Comparisons

Dependent Variable		(I) TR	(J) TR	Mean Difference (I-J)	Std. Error	Sig.	95% Confidence Interval Lower Bound	Upper Bound
DAY0	Tukey HSD	1,00	2,00	,22880*	,00562	,000	,2116	,2460
			3,00	,22880*	,00562	,000	,2116	,2460
		2,00	1,00	-,22880*	,00562	,000	-,2460	-,2116
			3,00	,00000	,00562	1,000	-,0172	,0172
		3,00	1,00	-,22880*	,00562	,000	-,2460	-,2116
			2,00	,00000	,00562	1,000	-,0172	,0172
	LSD	1,00	2,00	,22880*	,00562	,000	,2151	,2425
			3,00	,22880*	,00562	,000	,2151	,2425
		2,00	1,00	-,22880*	,00562	,000	-,2425	-,2151
			3,00	,00000	,00562	1,000	-,0137	,0137
		3,00	1,00	-,22880*	,00562	,000	-,2425	-,2151
			2,00	,00000	,00562	1,000	-,0137	,0137
	Bonferroni	1,00	2,00	,22880*	,00562	,000	,2103	,2473
			3,00	,22880*	,00562	,000	,2103	,2473
		2,00	1,00	-,22880*	,00562	,000	-,2473	-,2103
			3,00	,00000	,00562	1,000	-,0185	,0185
		3,00	1,00	-,22880*	,00562	,000	-,2473	-,2103
			2,00	,00000	,00562	1,000	-,0185	,0185
DAY2	Tukey HSD	1,00	2,00	,04420*	,01082	,015	,0110	,0774
			3,00	,09620*	,01082	,000	,0630	,1294
		2,00	1,00	-,04420*	,01082	,015	-,0774	-,0110
			3,00	,05200*	,01082	,007	,0188	,0852
		3,00	1,00	-,09620*	,01082	,000	-,1294	-,0630
			2,00	-,05200*	,01082	,007	-,0852	-,0188
	LSD	1,00	2,00	,04420*	,01082	,006	,0177	,0707
			3,00	,09620*	,01082	,000	,0697	,1227
		2,00	1,00	-,04420*	,01082	,006	-,0707	-,0177
			3,00	,05200*	,01082	,003	,0255	,0785
		3,00	1,00	-,09620*	,01082	,000	-,1227	-,0697
			2,00	-,05200*	,01082	,003	-,0785	-,0255
	Bonferroni	1,00	2,00	,04420*	,01082	,019	,0086	,0798
			3,00	,09620*	,01082	,000	,0606	,1318
		2,00	1,00	-,04420*	,01082	,019	-,0798	-,0086
			3,00	,05200*	,01082	,009	,0164	,0876
		3,00	1,00	-,09620*	,01082	,000	-,1318	-,0606
			2,00	-,05200*	,01082	,009	-,0876	-,0164
DAY4	Tukey HSD	1,00	2,00	,05460*	,01163	,008	,0189	,0903
			3,00	,13000*	,01163	,000	,0943	,1657
		2,00	1,00	-,05460*	,01163	,008	-,0903	-,0189
			3,00	,07540*	,01163	,002	,0397	,1111
		3,00	1,00	-,13000*	,01163	,000	-,1657	-,0943
			2,00	-,07540*	,01163	,002	-,1111	-,0397
	LSD	1,00	2,00	,05460*	,01163	,003	,0261	,0831
			3,00	,13000*	,01163	,000	,1015	,1585

		2,00	1,00	-,05460*	,01163	,003	-,0831	-,0261
			3,00	,07540*	,01163	,001	,0469	,1039
		3,00	1,00	-,13000*	,01163	,000	-,1585	-,1015
			2,00	-,07540*	,01163	,001	-,1039	-,0469
	Bonferroni	1,00	2,00	,05460*	,01163	,010	,0164	,0928
			3,00	,13000*	,01163	,000	,0918	,1682
		2,00	1,00	-,05460*	,01163	,010	-,0928	-,0164
			3,00	,07540*	,01163	,002	,0372	,1136
		3,00	1,00	-,13000*	,01163	,000	-,1682	-,0918
			2,00	-,07540*	,01163	,002	-,1136	-,0372
DAY6	Tukey HSD	1,00	2,00	,10140*	,01516	,001	,0549	,1479
			3,00	,19500*	,01516	,000	,1485	,2415
		2,00	1,00	-,10140*	,01516	,001	-,1479	-,0549
			3,00	,09360*	,01516	,002	,0471	,1401
		3,00	1,00	-,19500*	,01516	,000	-,2415	-,1485
			2,00	-,09360*	,01516	,002	-,1401	-,0471
	LSD	1,00	2,00	,10140*	,01516	,001	,0643	,1385
			3,00	,19500*	,01516	,000	,1579	,2321
		2,00	1,00	-,10140*	,01516	,001	-,1385	-,0643
			3,00	,09360*	,01516	,001	,0565	,1307
		3,00	1,00	-,19500*	,01516	,000	-,2321	-,1579
			2,00	-,09360*	,01516	,001	-,1307	-,0565
	Bonferroni	1,00	2,00	,10140*	,01516	,002	,0516	,1512
			3,00	,19500*	,01516	,000	,1452	,2448
		2,00	1,00	-,10140*	,01516	,002	-,1512	-,0516
			3,00	,09360*	,01516	,002	,0438	,1434
		3,00	1,00	-,19500*	,01516	,000	-,2448	-,1452
			2,00	-,09360*	,01516	,002	-,1434	-,0438
DAY8	Tukey HSD	1,00	2,00	,15340*	,01712	,000	,1009	,2059
			3,00	,22620*	,01712	,000	,1737	,2787
		2,00	1,00	-,15340*	,01712	,000	-,2059	-,1009
			3,00	,07280*	,01712	,013	,0203	,1253
		3,00	1,00	-,22620*	,01712	,000	-,2787	-,1737
			2,00	-,07280*	,01712	,013	-,1253	-,0203
	LSD	1,00	2,00	,15340*	,01712	,000	,1115	,1953
			3,00	,22620*	,01712	,000	,1843	,2681
		2,00	1,00	-,15340*	,01712	,000	-,1953	-,1115
			3,00	,07280*	,01712	,005	,0309	,1147
		3,00	1,00	-,22620*	,01712	,000	-,2681	-,1843
			2,00	-,07280*	,01712	,005	-,1147	-,0309
	Bonferroni	1,00	2,00	,15340*	,01712	,000	,0971	,2097
			3,00	,22620*	,01712	,000	,1699	,2825
		2,00	1,00	-,15340*	,01712	,000	-,2097	-,0971
			3,00	,07280*	,01712	,016	,0165	,1291
		3,00	1,00	-,22620*	,01712	,000	-,2825	-,1699

			2,00	-,07280*	,01712	,016	-,1291	-,0165
DAY10	Tukey HSD	1,00	2,00	,11440*	,02316	,006	,0433	,1855
			3,00	,26780*	,02316	,000	,1967	,3389
		2,00	1,00	-,11440*	,02316	,006	-,1855	-,0433
			3,00	,15340*	,02316	,001	,0823	,2245
		3,00	1,00	-,26780*	,02316	,000	-,3389	-,1967
			2,00	-,15340*	,02316	,001	-,2245	-,0823
	LSD	1,00	2,00	,11440*	,02316	,003	,0577	,1711
			3,00	,26780*	,02316	,000	,2111	,3245
		2,00	1,00	-,11440*	,02316	,003	-,1711	-,0577
			3,00	,15340*	,02316	,001	,0967	,2101
		3,00	1,00	-,26780*	,02316	,000	-,3245	-,2111
			2,00	-,15340*	,02316	,001	-,2101	-,0967
	Bonferroni	1,00	2,00	,11440*	,02316	,008	,0383	,1905
			3,00	,26780*	,02316	,000	,1917	,3439
		2,00	1,00	-,11440*	,02316	,008	-,1905	-,0383
			3,00	,15340*	,02316	,002	,0773	,2295
		3,00	1,00	-,26780*	,02316	,000	-,3439	-,1917
			2,00	-,15340*	,02316	,002	-,2295	-,0773
DAY12	Tukey HSD	1,00	2,00	,08060*	,01309	,002	,0404	,1208
			3,00	,22360*	,01309	,000	,1834	,2638
		2,00	1,00	-,08060*	,01309	,002	-,1208	-,0404
			3,00	,14300*	,01309	,000	,1028	,1832
		3,00	1,00	-,22360*	,01309	,000	-,2638	-,1834
			2,00	-,14300*	,01309	,000	-,1832	-,1028
	LSD	1,00	2,00	,08060*	,01309	,001	,0486	,1126
			3,00	,22360*	,01309	,000	,1916	,2556
		2,00	1,00	-,08060*	,01309	,001	-,1126	-,0486
			3,00	,14300*	,01309	,000	,1110	,1750
		3,00	1,00	-,22360*	,01309	,000	-,2556	-,1916
			2,00	-,14300*	,01309	,000	-,1750	-,1110
	Bonferroni	1,00	2,00	,08060*	,01309	,003	,0376	,1236
			3,00	,22360*	,01309	,000	,1806	,2666
		2,00	1,00	-,08060*	,01309	,003	-,1236	-,0376
			3,00	,14300*	,01309	,000	,1000	,1860
		3,00	1,00	-,22360*	,01309	,000	-,2666	-,1806
			2,00	-,14300*	,01309	,000	-,1860	-,1000

*. The mean difference is significant at the 0.05 level. TR: Packaging treatment, 1:LDPE, 2:LDPE-AC, 3:LDPE-AC-TO

Table S5. Multiple comparison tests of heme iron values of pork meat during storage with respect to packaging treatment

Multiple Comparisons

Dependent Variable		(I) TR	(J) TR	Mean Difference (I-J)	Std. Error	Sig.	95% Confidence Interval Lower Bound	Upper Bound
DAY0	Tukey HSD	1,00	2,00	10,17593*	,05885	,000	9,9953	10,3565

			3,00	10,17593*	,05885	,000	9,9953	10,3565
		2,00	1,00	-10,17593*	,05885	,000	-10,3565	-9,9953
			3,00	,00000	,05885	1,000	-,1806	,1806
		3,00	1,00	-10,17593*	,05885	,000	-10,3565	-9,9953
			2,00	,00000	,05885	1,000	-,1806	,1806
	LSD	1,00	2,00	10,17593*	,05885	,000	10,0319	10,3199
			3,00	10,17593*	,05885	,000	10,0319	10,3199
		2,00	1,00	-10,17593*	,05885	,000	-10,3199	-10,0319
			3,00	,00000	,05885	1,000	-,1440	,1440
		3,00	1,00	-10,17593*	,05885	,000	-10,3199	-10,0319
			2,00	,00000	,05885	1,000	-,1440	,1440
	Bonferroni	1,00	2,00	10,17593*	,05885	,000	9,9824	10,3694
			3,00	10,17593*	,05885	,000	9,9824	10,3694
		2,00	1,00	-10,17593*	,05885	,000	-10,3694	-9,9824
			3,00	,00000	,05885	1,000	-,1935	,1935
		3,00	1,00	-10,17593*	,05885	,000	-10,3694	-9,9824
			2,00	,00000	,05885	1,000	-,1935	,1935
DAY2	Tukey HSD	1,00	2,00	-,95962*	,09234	,000	-1,2429	-,6763
			3,00	-1,65934*	,09234	,000	-1,9427	-1,3760
		2,00	1,00	,95962*	,09234	,000	,6763	1,2429
			3,00	-,69972*	,09234	,001	-,9830	-,4164
		3,00	1,00	1,65934*	,09234	,000	1,3760	1,9427
			2,00	,69972*	,09234	,001	,4164	,9830
	LSD	1,00	2,00	-,95962*	,09234	,000	-1,1856	-,7337
			3,00	-1,65934*	,09234	,000	-1,8853	-1,4334
		2,00	1,00	,95962*	,09234	,000	,7337	1,1856
			3,00	-,69972*	,09234	,000	-,9257	-,4738
		3,00	1,00	1,65934*	,09234	,000	1,4334	1,8853
			2,00	,69972*	,09234	,000	,4738	,9257
	Bonferroni	1,00	2,00	-,95962*	,09234	,000	-1,2632	-,6561
			3,00	-1,65934*	,09234	,000	-1,9629	-1,3558
		2,00	1,00	,95962*	,09234	,000	,6561	1,2632
			3,00	-,69972*	,09234	,001	-1,0033	-,3962
		3,00	1,00	1,65934*	,09234	,000	1,3558	1,9629
			2,00	,69972*	,09234	,001	,3962	1,0033
DAY4	Tukey HSD	1,00	2,00	-,63974*	,09234	,001	-,9231	-,3564
			3,00	-1,75930*	,09234	,000	-2,0426	-1,4760
		2,00	1,00	,63974*	,09234	,001	,3564	,9231
			3,00	-1,11955*	,09234	,000	-1,4029	-,8362
		3,00	1,00	1,75930*	,09234	,000	1,4760	2,0426
			2,00	1,11955*	,09234	,000	,8362	1,4029
	LSD	1,00	2,00	-,63974*	,09234	,000	-,8657	-,4138
			3,00	-1,75930*	,09234	,000	-1,9852	-1,5334
		2,00	1,00	,63974*	,09234	,000	,4138	,8657
			3,00	-1,11955*	,09234	,000	-1,3455	-,8936

		3,00	1,00	1,75930*	,09234	,000	1,5334	1,9852
			2,00	1,11955*	,09234	,000	,8936	1,3455
	Bonferroni	1,00	2,00	-,63974*	,09234	,001	-,9433	-,3362
			3,00	-1,75930*	,09234	,000	-2,0629	-1,4557
		2,00	1,00	,63974*	,09234	,001	,3362	,9433
			3,00	-1,11955*	,09234	,000	-1,4231	-,8160
		3,00	1,00	1,75930*	,09234	,000	1,4557	2,0629
			2,00	1,11955*	,09234	,000	,8160	1,4231
DAY6	Tukey HSD	1,00	2,00	-,59976*	,16727	,027	-1,1130	-,0865
			3,00	-2,27909*	,16727	,000	-2,7923	-1,7659
		2,00	1,00	,59976*	,16727	,027	,0865	1,1130
			3,00	-1,67933*	,16727	,000	-2,1925	-1,1661
		3,00	1,00	2,27909*	,16727	,000	1,7659	2,7923
			2,00	1,67933*	,16727	,000	1,1661	2,1925
	LSD	1,00	2,00	-,59976*	,16727	,012	-1,0090	-,1905
			3,00	-2,27909*	,16727	,000	-2,6884	-1,8698
		2,00	1,00	,59976*	,16727	,012	,1905	1,0090
			3,00	-1,67933*	,16727	,000	-2,0886	-1,2700
		3,00	1,00	2,27909*	,16727	,000	1,8698	2,6884
			2,00	1,67933*	,16727	,000	1,2700	2,0886
	Bonferroni	1,00	2,00	-,59976*	,16727	,035	-1,1496	-,0499
			3,00	-2,27909*	,16727	,000	-2,8290	-1,7292
		2,00	1,00	,59976*	,16727	,035	,0499	1,1496
			3,00	-1,67933*	,16727	,000	-2,2292	-1,1295
		3,00	1,00	2,27909*	,16727	,000	1,7292	2,8290
			2,00	1,67933*	,16727	,000	1,1295	2,2292
DAY8	Tukey HSD	1,00	2,00	-,59976*	,13559	,011	-1,0158	-,1837
			3,00	-2,15914*	,13559	,000	-2,5752	-1,7431
		2,00	1,00	,59976*	,13559	,011	,1837	1,0158
			3,00	-1,55938*	,13559	,000	-1,9754	-1,1433
		3,00	1,00	2,15914*	,13559	,000	1,7431	2,5752
			2,00	1,55938*	,13559	,000	1,1433	1,9754
	LSD	1,00	2,00	-,59976*	,13559	,004	-,9315	-,2680
			3,00	-2,15914*	,13559	,000	-2,4909	-1,8274
		2,00	1,00	,59976*	,13559	,004	,2680	,9315
			3,00	-1,55938*	,13559	,000	-1,8912	-1,2276
		3,00	1,00	2,15914*	,13559	,000	1,8274	2,4909
			2,00	1,55938*	,13559	,000	1,2276	1,8912
	Bonferroni	1,00	2,00	-,59976*	,13559	,013	-1,0455	-,1540
			3,00	-2,15914*	,13559	,000	-2,6049	-1,7134
		2,00	1,00	,59976*	,13559	,013	,1540	1,0455
			3,00	-1,55938*	,13559	,000	-2,0051	-1,1136
		3,00	1,00	2,15914*	,13559	,000	1,7134	2,6049
			2,00	1,55938*	,13559	,000	1,1136	2,0051
DAY10	Tukey HSD	1,00	2,00	-,25990	,17042	,345	-,7828	,2630

			3,00	-1,61935*	,17042	,000	-2,1423	-1,0965
		2,00	1,00	,25990	,17042	,345	-,2630	,7828
			3,00	-1,35946*	,17042	,001	-1,8824	-,8366
		3,00	1,00	1,61935*	,17042	,000	1,0965	2,1423
			2,00	1,35946*	,17042	,001	,8366	1,8824
	LSD	1,00	2,00	-,25990	,17042	,178	-,6769	,1571
			3,00	-1,61935*	,17042	,000	-2,0364	-1,2023
		2,00	1,00	,25990	,17042	,178	-,1571	,6769
			3,00	-1,35946*	,17042	,000	-1,7765	-,9425
		3,00	1,00	1,61935*	,17042	,000	1,2023	2,0364
			2,00	1,35946*	,17042	,000	,9425	1,7765
	Bonferroni	1,00	2,00	-,25990	,17042	,534	-,8201	,3004
			3,00	-1,61935*	,17042	,000	-2,1796	-1,0591
		2,00	1,00	,25990	,17042	,534	-,3004	,8201
			3,00	-1,35946*	,17042	,001	-1,9197	-,7992
		3,00	1,00	1,61935*	,17042	,000	1,0591	2,1796
			2,00	1,35946*	,17042	,001	,7992	1,9197
DAY12	Tukey HSD	1,00	2,00	-,53978*	,10950	,006	-,8758	-,2038
			3,00	-1,19952*	,10950	,000	-1,5355	-,8635
		2,00	1,00	,53978*	,10950	,006	,2038	,8758
			3,00	-,65974*	,10950	,002	-,9957	-,3238
		3,00	1,00	1,19952*	,10950	,000	,8635	1,5355
			2,00	,65974*	,10950	,002	,3238	,9957
	LSD	1,00	2,00	-,53978*	,10950	,003	-,8077	-,2718
			3,00	-1,19952*	,10950	,000	-1,4675	-,9316
		2,00	1,00	,53978*	,10950	,003	,2718	,8077
			3,00	-,65974*	,10950	,001	-,9277	-,3918
		3,00	1,00	1,19952*	,10950	,000	,9316	1,4675
			2,00	,65974*	,10950	,001	,3918	,9277
	Bonferroni	1,00	2,00	-,53978*	,10950	,008	-,8998	-,1798
			3,00	-1,19952*	,10950	,000	-1,5595	-,8395
		2,00	1,00	,53978*	,10950	,008	,1798	,8998
			3,00	-,65974*	,10950	,003	-1,0197	-,2998
		3,00	1,00	1,19952*	,10950	,000	,8395	1,5595
			2,00	,65974*	,10950	,003	,2998	1,0197

*. The mean difference is significant at the 0.05 level.TR: Packaging treatment, 1:LDPE, 2:LDPE-AC, 3:LDPE-AC-TO

Table S6. Pearson's correlation of heme iron with TBARS values of pork meat during storage within each packaging treatments

Correlations

		DAY0	DAY2	DAY4	DAY6	DAY8	DAY10	DAY12
DAY0	Pearson Correlation	1	,346	,326	,275	,198	,103	-,269
	Sig. (2-tailed)		,160	,187	,269	,432	,685	,281
	N	18	18	18	18	18	18	18

DAY2	Pearson Correlation	,346	1	,998**	,989**	,969**	,903**	,719**
	Sig. (2-tailed)	,160		,000	,000	,000	,000	,001
	N	18	18	18	18	18	18	18
DAY4	Pearson Correlation	,326	,998**	1	,995**	,980**	,923**	,749**
	Sig. (2-tailed)	,187	,000		,000	,000	,000	,000
	N	18	18	18	18	18	18	18
DAY6	Pearson Correlation	,275	,989**	,995**	1	,991**	,954**	,794**
	Sig. (2-tailed)	,269	,000	,000		,000	,000	,000
	N	18	18	18	18	18	18	18
DAY8	Pearson Correlation	,198	,969**	,980**	,991**	1	,973**	,846**
	Sig. (2-tailed)	,432	,000	,000	,000		,000	,000
	N	18	18	18	18	18	18	18
DAY10	Pearson Correlation	,103	,903**	,923**	,954**	,973**	1	,902**
	Sig. (2-tailed)	,685	,000	,000	,000	,000		,000
	N	18	18	18	18	18	18	18
DAY12	Pearson Correlation	-,269	,719**	,749**	,794**	,846**	,902**	1
	Sig. (2-tailed)	,281	,001	,000	,000	,000	,000	
	N	18	18	18	18	18	18	18

**. Correlation is significant at the 0.01 level (2-tailed).

Table S7. ANOVA Results of TVC.

ANOVA

				Sum of Squares	df	Mean Square	F	Sig.
DAY0	Between Groups	(Combined)		,000	2	,000	,000	1,000
		Linear Term	Contrast	,000	1	,000	,000	1,000
			Deviation	,000	1	,000	,000	1,000
	Within Groups			,022	6	,004		
	Total			,022	8			
DAY2	Between Groups	(Combined)		,742	2	,371	8,914	,016
		Linear Term	Contrast	,735	1	,735	17,654	,006
			Deviation	,007	1	,007	,173	,692
	Within Groups			,250	6	,042		
	Total			,992	8			
DAY4	Between Groups	(Combined)		2,251	2	1,126	35,032	,000
		Linear Term	Contrast	1,882	1	1,882	58,556	,000
			Deviation	,370	1	,370	11,508	,015
	Within Groups			,193	6	,032		
	Total			2,444	8			
DAY6	Between Groups	(Combined)		2,382	2	1,191	11,935	,008
		Linear Term	Contrast	2,196	1	2,196	22,006	,003
			Deviation	,186	1	,186	1,864	,221
	Within Groups			,599	6	,100		
	Total			2,981	8			
DAY8	Between Groups	(Combined)		1,680	2	,840	28,478	,001
		Linear Term	Contrast	1,622	1	1,622	54,997	,000
			Deviation	,058	1	,058	1,959	,211
	Within Groups			,177	6	,030		
	Total			1,857	8			

** 1 = LDPE, 2 = LDPE-AC, 3 = LDPE-AC-TO.

Table S8 Multiple Comparisons tests.

Multiple Comparisons

Dependent Variable		(I) TO	(J) TO	Mean Difference (I-J)	Std. Error	Sig.	95% Confidence Interval Lower Bound	Upper Bound
DAY0	Tukey HSD	1,00	2,00	,00000	,04899	1,000	-,1503	,1503
			3,00	,00000	,04899	1,000	-,1503	,1503
		2,00	1,00	,00000	,04899	1,000	-,1503	,1503
			3,00	,00000	,04899	1,000	-,1503	,1503
		3,00	1,00	,00000	,04899	1,000	-,1503	,1503
			2,00	,00000	,04899	1,000	-,1503	,1503
	LSD	1,00	2,00	,00000	,04899	1,000	-,1199	,1199
			3,00	,00000	,04899	1,000	-,1199	,1199
		2,00	1,00	,00000	,04899	1,000	-,1199	,1199
			3,00	,00000	,04899	1,000	-,1199	,1199
		3,00	1,00	,00000	,04899	1,000	-,1199	,1199
			2,00	,00000	,04899	1,000	-,1199	,1199
	Bonferroni	1,00	2,00	,00000	,04899	1,000	-,1611	,1611
			3,00	,00000	,04899	1,000	-,1611	,1611
		2,00	1,00	,00000	,04899	1,000	-,1611	,1611
			3,00	,00000	,04899	1,000	-,1611	,1611
		3,00	1,00	,00000	,04899	1,000	-,1611	,1611
			2,00	,00000	,04899	1,000	-,1611	,1611
DAY2	Tukey HSD	1,00	2,00	,41000	,16660	,107	-,1012	,9212
			3,00	,70000*	,16660	,013	,1888	1,2112
		2,00	1,00	-,41000	,16660	,107	-,9212	,1012
			3,00	,29000	,16660	,267	-,2212	,8012
		3,00	1,00	-,70000*	,16660	,013	-1,2112	-,1888
			2,00	-,29000	,16660	,267	-,8012	,2212
	LSD	1,00	2,00	,41000*	,16660	,049	,0023	,8177
			3,00	,70000*	,16660	,006	,2923	1,1077
		2,00	1,00	-,41000*	,16660	,049	-,8177	-,0023
			3,00	,29000	,16660	,132	-,1177	,6977
		3,00	1,00	-,70000*	,16660	,006	-1,1077	-,2923
			2,00	-,29000	,16660	,132	-,6977	,1177
	Bonferroni	1,00	2,00	,41000	,16660	,147	-,1377	,9577
			3,00	,70000*	,16660	,017	,1523	1,2477
		2,00	1,00	-,41000	,16660	,147	-,9577	,1377
			3,00	,29000	,16660	,397	-,2577	,8377
		3,00	1,00	-,70000*	,16660	,017	-1,2477	-,1523
			2,00	-,29000	,16660	,397	-,8377	,2577
DAY4	Tukey HSD	1,00	2,00	,13000	,14636	,667	-,3191	,5791
			3,00	1,12000*	,14636	,001	,6709	1,5691
		2,00	1,00	-,13000	,14636	,667	-,5791	,3191

			3,00	,99000*	,14636	,001	,5409	1,4391
		3,00	1,00	-1,12000*	,14636	,001	-1,5691	-,6709
			2,00	-,99000*	,14636	,001	-1,4391	-,5409
	LSD	1,00	2,00	,13000	,14636	,409	-,2281	,4881
			3,00	1,12000*	,14636	,000	,7619	1,4781
		2,00	1,00	-,13000	,14636	,409	-,4881	,2281
			3,00	,99000*	,14636	,001	,6319	1,3481
		3,00	1,00	-1,12000*	,14636	,000	-1,4781	-,7619
			2,00	-,99000*	,14636	,001	-1,3481	-,6319
	Bonferroni	1,00	2,00	,13000	,14636	1,000	-,3512	,6112
			3,00	1,12000*	,14636	,001	,6388	1,6012
		2,00	1,00	-,13000	,14636	1,000	-,6112	,3512
			3,00	,99000*	,14636	,002	,5088	1,4712
		3,00	1,00	-1,12000*	,14636	,001	-1,6012	-,6388
			2,00	-,99000*	,14636	,002	-1,4712	-,5088
DAY6	Tukey HSD	1,00	2,00	,30000	,25794	,515	-,4914	1,0914
			3,00	1,21000*	,25794	,008	,4186	2,0014
		2,00	1,00	-,30000	,25794	,515	-1,0914	,4914
			3,00	,91000*	,25794	,029	,1186	1,7014
		3,00	1,00	-1,21000*	,25794	,008	-2,0014	-,4186
			2,00	-,91000*	,25794	,029	-1,7014	-,1186
	LSD	1,00	2,00	,30000	,25794	,289	-,3312	,9312
			3,00	1,21000*	,25794	,003	,5788	1,8412
		2,00	1,00	-,30000	,25794	,289	-,9312	,3312
			3,00	,91000*	,25794	,012	,2788	1,5412
		3,00	1,00	-1,21000*	,25794	,003	-1,8412	-,5788
			2,00	-,91000*	,25794	,012	-1,5412	-,2788
	Bonferroni	1,00	2,00	,30000	,25794	,867	-,5480	1,1480
			3,00	1,21000*	,25794	,010	,3620	2,0580
		2,00	1,00	-,30000	,25794	,867	-1,1480	,5480
			3,00	,91000*	,25794	,037	,0620	1,7580
		3,00	1,00	-1,21000*	,25794	,010	-2,0580	-,3620
			2,00	-,91000*	,25794	,037	-1,7580	-,0620
DAY8	Tukey HSD	1,00	2,00	,35000	,14024	,103	-,0803	,7803
			3,00	1,04000*	,14024	,001	,6097	1,4703
		2,00	1,00	-,35000	,14024	,103	-,7803	,0803
			3,00	,69000*	,14024	,006	,2597	1,1203
		3,00	1,00	-1,04000*	,14024	,001	-1,4703	-,6097
			2,00	-,69000*	,14024	,006	-1,1203	-,2597
	LSD	1,00	2,00	,35000*	,14024	,047	,0069	,6931
			3,00	1,04000*	,14024	,000	,6969	1,3831
		2,00	1,00	-,35000*	,14024	,047	-,6931	-,0069
			3,00	,69000*	,14024	,003	,3469	1,0331
		3,00	1,00	-1,04000*	,14024	,000	-1,3831	-,6969
			2,00	-,69000*	,14024	,003	-1,0331	-,3469

	Bonferroni	1,00	2,00	,35000	,14024	,140	-,1110	,8110
			3,00	1,04000*	,14024	,001	,5790	1,5010
		2,00	1,00	-,35000	,14024	,140	-,8110	,1110
			3,00	,69000*	,14024	,008	,2290	1,1510
		3,00	1,00	-1,04000*	,14024	,001	-1,5010	-,5790
			2,00	-,69000*	,14024	,008	-1,1510	-,2290

*. The mean difference is significant at the 0.05 level. ** 1 = LDPE, 2 = LDPE/15AC, 3 = LDPE/15TO@AC.

Table S9. ANOVA ODOR

Multiple Comparisons

Dependent Variable		(I) TO	(J) TO	Mean Difference (I-J)	Std. Error	Sig.	95% Confidence Interval	
							Lower Bound	Upper Bound
DAY2	Tukey HSD	1,00	2,00	,00000	,17243	1,000	-,4600	,4600
			3,00	-,16000	,17243	,634	-,6200	,3000
		2,00	1,00	,00000	,17243	1,000	-,4600	,4600
			3,00	-,16000	,17243	,634	-,6200	,3000
		3,00	1,00	,16000	,17243	,634	-,3000	,6200
			2,00	,16000	,17243	,634	-,3000	,6200
	LSD	1,00	2,00	,00000	,17243	1,000	-,3757	,3757
			3,00	-,16000	,17243	,372	-,5357	,2157
		2,00	1,00	,00000	,17243	1,000	-,3757	,3757
			3,00	-,16000	,17243	,372	-,5357	,2157
		3,00	1,00	,16000	,17243	,372	-,2157	,5357
			2,00	,16000	,17243	,372	-,2157	,5357
	Bonferroni	1,00	2,00	,00000	,17243	1,000	-,4793	,4793
			3,00	-,16000	,17243	1,000	-,6393	,3193
		2,00	1,00	,00000	,17243	1,000	-,4793	,4793
			3,00	-,16000	,17243	1,000	-,6393	,3193
		3,00	1,00	,16000	,17243	1,000	-,3193	,6393
			2,00	,16000	,17243	1,000	-,3193	,6393

DAY4	Tukey HSD	1,00	2,00	,00000	,27105	1,000	-,7231	,7231
			3,00	-,34000	,27105	,446	-1,0631	,3831
		2,00	1,00	,00000	,27105	1,000	-,7231	,7231
			3,00	-,34000	,27105	,446	-1,0631	,3831
		3,00	1,00	,34000	,27105	,446	-,3831	1,0631
			2,00	,34000	,27105	,446	-,3831	1,0631
	LSD	1,00	2,00	,00000	,27105	1,000	-,5906	,5906
			3,00	-,34000	,27105	,234	-,9306	,2506
		2,00	1,00	,00000	,27105	1,000	-,5906	,5906
			3,00	-,34000	,27105	,234	-,9306	,2506
		3,00	1,00	,34000	,27105	,234	-,2506	,9306
			2,00	,34000	,27105	,234	-,2506	,9306
	Bonferroni	1,00	2,00	,00000	,27105	1,000	-,7534	,7534
			3,00	-,34000	,27105	,701	-1,0934	,4134
		2,00	1,00	,00000	,27105	1,000	-,7534	,7534
			3,00	-,34000	,27105	,701	-1,0934	,4134
		3,00	1,00	,34000	,27105	,701	-,4134	1,0934
			2,00	,34000	,27105	,701	-,4134	1,0934
DAY6	Tukey HSD	1,00	2,00	-,22000	,16411	,401	-,6578	,2178
			3,00	-,70000*	,16411	,003	-1,1378	-,2622
		2,00	1,00	,22000	,16411	,401	-,2178	,6578
			3,00	-,48000*	,16411	,032	-,9178	-,0422
		3,00	1,00	,70000*	,16411	,003	,2622	1,1378
			2,00	,48000*	,16411	,032	,0422	,9178
	LSD	1,00	2,00	-,22000	,16411	,205	-,5776	,1376
			3,00	-,70000*	,16411	,001	-1,0576	-,3424
		2,00	1,00	,22000	,16411	,205	-,1376	,5776
			3,00	-,48000*	,16411	,013	-,8376	-,1224
		3,00	1,00	,70000*	,16411	,001	,3424	1,0576
			2,00	,48000*	,16411	,013	,1224	,8376

	Bonferroni	1,00	2,00	-,22000	,16411	,615	-,6761	,2361
			3,00	-,70000*	,16411	,003	-1,1561	-,2439
		2,00	1,00	,22000	,16411	,615	-,2361	,6761
			3,00	-,48000*	,16411	,038	-,9361	-,0239
		3,00	1,00	,70000*	,16411	,003	,2439	1,1561
			2,00	,48000*	,16411	,038	,0239	,9361

*. The mean difference is significant at the 0.05 level.

Table S10. ANOVA COLOR

Multiple Comparisons

Dependent Variable		(I) TO	(J) TO	Mean Difference (I-J)	Std. Error	Sig.	95% Confidence Interval	
							Lower Bound	Upper Bound
DAY2	Tukey HSD	1,00	2,00	,26000	,21479	,470	-,3130	,8330
			3,00	,00000	,21479	1,000	-,5730	,5730
		2,00	1,00	-,26000	,21479	,470	-,8330	,3130
			3,00	-,26000	,21479	,470	-,8330	,3130
		3,00	1,00	,00000	,21479	1,000	-,5730	,5730
			2,00	,26000	,21479	,470	-,3130	,8330
	LSD	1,00	2,00	,26000	,21479	,249	-,2080	,7280
			3,00	,00000	,21479	1,000	-,4680	,4680
		2,00	1,00	-,26000	,21479	,249	-,7280	,2080
			3,00	-,26000	,21479	,249	-,7280	,2080
		3,00	1,00	,00000	,21479	1,000	-,4680	,4680
			2,00	,26000	,21479	,249	-,2080	,7280
	Bonferroni	1,00	2,00	,26000	,21479	,748	-,3370	,8570
			3,00	,00000	,21479	1,000	-,5970	,5970
		2,00	1,00	-,26000	,21479	,748	-,8570	,3370

			3,00	-,26000	,21479	,748	-,8570	,3370
		3,00	1,00	,00000	,21479	1,000	-,5970	,5970
			2,00	,26000	,21479	,748	-,3370	,8570
DAY4	Tukey HSD	1,00	2,00	,08000	,31027	,964	-,7478	,9078
			3,00	-,22000	,31027	,763	-1,0478	,6078
		2,00	1,00	-,08000	,31027	,964	-,9078	,7478
			3,00	-,30000	,31027	,610	-1,1278	,5278
		3,00	1,00	,22000	,31027	,763	-,6078	1,0478
			2,00	,30000	,31027	,610	-,5278	1,1278
	LSD	1,00	2,00	,08000	,31027	,801	-,5960	,7560
			3,00	-,22000	,31027	,492	-,8960	,4560
		2,00	1,00	-,08000	,31027	,801	-,7560	,5960
			3,00	-,30000	,31027	,353	-,9760	,3760
		3,00	1,00	,22000	,31027	,492	-,4560	,8960
			2,00	,30000	,31027	,353	-,3760	,9760
	Bonferroni	1,00	2,00	,08000	,31027	1,000	-,7824	,9424
			3,00	-,22000	,31027	1,000	-1,0824	,6424
		2,00	1,00	-,08000	,31027	1,000	-,9424	,7824
			3,00	-,30000	,31027	1,000	-1,1624	,5624
		3,00	1,00	,22000	,31027	1,000	-,6424	1,0824
			2,00	,30000	,31027	1,000	-,5624	1,1624
DAY6	Tukey HSD	1,00	2,00	-,24000	,10646	,102	-,5240	,0440
			3,00	-1,06000*	,10646	,000	-1,3440	-,7760
		2,00	1,00	,24000	,10646	,102	-,0440	,5240
			3,00	-,82000*	,10646	,000	-1,1040	-,5360
		3,00	1,00	1,06000*	,10646	,000	,7760	1,3440
			2,00	,82000*	,10646	,000	,5360	1,1040
	LSD	1,00	2,00	-,24000*	,10646	,044	-,4720	-,0080
			3,00	-1,06000*	,10646	,000	-1,2920	-,8280
		2,00	1,00	,24000*	,10646	,044	,0080	,4720

			3,00	-,82000*	,10646	,000	-1,0520	-,5880
		3,00	1,00	1,06000*	,10646	,000	,8280	1,2920
			2,00	,82000*	,10646	,000	,5880	1,0520
	Bonferroni	1,00	2,00	-,24000	,10646	,131	-,5359	,0559
			3,00	-1,06000*	,10646	,000	-1,3559	-,7641
		2,00	1,00	,24000	,10646	,131	-,0559	,5359
			3,00	-,82000*	,10646	,000	-1,1159	-,5241
		3,00	1,00	1,06000*	,10646	,000	,7641	1,3559
			2,00	,82000*	,10646	,000	,5241	1,1159

*. The mean difference is significant at the 0.05 level.

Table S11. ANOVA COHESION

Multiple Comparisons

Dependent Variable		(I) TO	(J) TO	Mean Difference (I-J)	Std. Error	Sig.	95% Confidence Interval	
							Lower Bound	Upper Bound
DAY2	Tukey HSD	1,00	2,00	,10000	,20559	,879	-,4485	,6485
			3,00	,02000	,20559	,995	-,5285	,5685
		2,00	1,00	-,10000	,20559	,879	-,6485	,4485
			3,00	-,08000	,20559	,920	-,6285	,4685
		3,00	1,00	-,02000	,20559	,995	-,5685	,5285
			2,00	,08000	,20559	,920	-,4685	,6285
	LSD	1,00	2,00	,10000	,20559	,635	-,3479	,5479
			3,00	,02000	,20559	,924	-,4279	,4679
		2,00	1,00	-,10000	,20559	,635	-,5479	,3479
			3,00	-,08000	,20559	,704	-,5279	,3679
		3,00	1,00	-,02000	,20559	,924	-,4679	,4279
			2,00	,08000	,20559	,704	-,3679	,5279
	Bonferroni	1,00	2,00	,10000	,20559	1,000	-,4714	,6714

			3,00	,02000	,20559	1,000	-,5514	,5914
		2,00	1,00	-,10000	,20559	1,000	-,6714	,4714
			3,00	-,08000	,20559	1,000	-,6514	,4914
		3,00	1,00	-,02000	,20559	1,000	-,5914	,5514
			2,00	,08000	,20559	1,000	-,4914	,6514
DAY4	Tukey HSD	1,00	2,00	-,34000	,26633	,434	-1,0505	,3705
			3,00	-,48000	,26633	,210	-1,1905	,2305
		2,00	1,00	,34000	,26633	,434	-,3705	1,0505
			3,00	-,14000	,26633	,860	-,8505	,5705
		3,00	1,00	,48000	,26633	,210	-,2305	1,1905
			2,00	,14000	,26633	,860	-,5705	,8505
	LSD	1,00	2,00	-,34000	,26633	,226	-,9203	,2403
			3,00	-,48000	,26633	,097	-1,0603	,1003
		2,00	1,00	,34000	,26633	,226	-,2403	,9203
			3,00	-,14000	,26633	,609	-,7203	,4403
		3,00	1,00	,48000	,26633	,097	-,1003	1,0603
			2,00	,14000	,26633	,609	-,4403	,7203
	Bonferroni	1,00	2,00	-,34000	,26633	,678	-1,0803	,4003
			3,00	-,48000	,26633	,290	-1,2203	,2603
		2,00	1,00	,34000	,26633	,678	-,4003	1,0803
			3,00	-,14000	,26633	1,000	-,8803	,6003
		3,00	1,00	,48000	,26633	,290	-,2603	1,2203
			2,00	,14000	,26633	1,000	-,6003	,8803
DAY6	Tukey HSD	1,00	2,00	-,60000*	,13614	,002	-,9632	-,2368
			3,00	-1,10000*	,13614	,000	-1,4632	-,7368
		2,00	1,00	,60000*	,13614	,002	,2368	,9632
			3,00	-,50000*	,13614	,008	-,8632	-,1368
		3,00	1,00	1,10000*	,13614	,000	,7368	1,4632
			2,00	,50000*	,13614	,008	,1368	,8632
	LSD	1,00	2,00	-,60000*	,13614	,001	-,8966	-,3034

			3,00	-1,10000*	,13614	,000	-1,3966	-,8034
		2,00	1,00	,60000*	,13614	,001	,3034	,8966
			3,00	-,50000*	,13614	,003	-,7966	-,2034
		3,00	1,00	1,10000*	,13614	,000	,8034	1,3966
			2,00	,50000*	,13614	,003	,2034	,7966
	Bonferroni	1,00	2,00	-,60000*	,13614	,003	-,9784	-,2216
			3,00	-1,10000*	,13614	,000	-1,4784	-,7216
		2,00	1,00	,60000*	,13614	,003	,2216	,9784
			3,00	-,50000*	,13614	,010	-,8784	-,1216
		3,00	1,00	1,10000*	,13614	,000	,7216	1,4784
			2,00	,50000*	,13614	,010	,1216	,8784

*. The mean difference is significant at the 0.05 level.

1,00: LDPE

2,00: LDPE/15AC

3,00: LDPE/15TO@AC
